# Using Two X-Ray Images to Create a Parameterized Scoliotic Spine Model and Analyze Disk Stress Adjacent to Spinal Fixation—A Finite Element Analysis

**DOI:** 10.3390/bioengineering12111212

**Published:** 2025-11-06

**Authors:** Te-Han Wang, Po-Hsing Chou, Chen-Sheng Chen

**Affiliations:** 1 Department of Physical Therapy and Assistive Technology, National Yang Ming Chiao Tung University, Taipei 112304, Taiwan; 2 Department of Orthopaedics and Traumatology, Taipei Veterans General Hospital, Taipei 112201, Taiwan; 3 Department of Orthopedics, School of Medicine, National Yang Ming Chiao Tung University, Taipei 112304, Taiwan

**Keywords:** scoliosis, finite element model, X-ray image, adjacent disk, biomechanics

## Abstract

Posterior instrumentation is used to treat severe adolescent idiopathic scoliosis (AIS) with a Cobb angle greater than 40 degrees. Clinical studies indicate that AIS patients may develop adjacent segment degeneration (ASD) post-surgery. However, there is limited research on the biomechanical effects on adjacent segments after surgery, and straightforward methods for creating finite element (FE) models that reflect vertebral deformation are lacking. Therefore, this study aims to use biplanar X-ray images to establish a case-specific, parameterized FE model reflecting coronal plane vertebral deformation and employ FE analysis to compare pre- and postoperative changes in the range of motion (ROM), endplate stress, and intervertebral disk stress of adjacent segments. We developed an FE model from biplanar X-ray images of a patient with AIS, using ANSYS software to establish pre- and postoperative models. The shape of the preoperative model was validated using computed tomography (CT) reconstruction. A flexion moment was applied to C7 of the spine model to achieve the same forward bending angle in the pre- and postoperative models. This study successfully developed a case-specific parameterized FE model based on X-ray images. The differences between Cobb angle and thoracolumbar kyphosis angle measurements in X-ray images and CT reconstructions were 6.5 and 5.4 mm. This FE model was used to analyze biomechanical effects on motion segments adjacent to the fixation site, revealing a decrease in maximum endplate and disk stress in the cranial segment and an increase in stress in the caudal segment.

## 1. Introduction

Adolescent idiopathic scoliosis (AIS) is a common spinal deformity with unknown etiology, characterized by spinal curvature along the coronal plane and associated spinal rotation. AIS typically affects adolescents aged 10 to 16 years, with an incidence rate of about 2–3% and a higher prevalence in female patients [1]. The severity of AIS is generally measured using the Cobb angle; when the angle exceeds 40 degrees, surgery is often recommended. Posterior instrumentation is the most common surgical method, using implanted metal devices to correct the abnormal curvature [2].

Despite the effectiveness of surgery in correcting scoliosis and improving patients’ quality of life, clinical research indicates that adjacent segment degeneration (ASD) may occur post-operatively [3,4,5,6]. Potential risk factors for ASD include longer fusion segments [3], lower fixation levels [4,5], greater residual curvature, and larger post-operative thoracolumbar kyphosis (TLK) angles [6,7]. ASD often presents as compensatory increased ROM in unfused adjacent segments, leading to increased stress on intervertebral disks and endplates [8,9], which results in disk degeneration, pain, and functional impairments. An uneven stress distribution may also cause Modic changes in endplates [5,6], as well as degenerative lesions affecting the endplates. Although clinical research suggests that patients with AIS are at risk of ASD post-surgery, it remains unclear whether the surgery itself increases the ASD risk and how biomechanical effects on adjacent segments change before and after surgery, necessitating further research.

Finite element (FE) analysis is crucial for studying spinal biomechanics, enabling simulations based on a patient-specific parameterized FE model. Currently, the application of FE analysis in the field of AIS primarily includes examining the stress imbalance caused by scoliosis, the effects of the brace, and surgical treatment [10,11,12,13,14,15]. To assess stress imbalance, Zhang et al. [10] used CT-based lumbar spine models to show that flexion stress accumulates on the concave side of the scoliotic curve; Li et al. [12] used CT-based spine models to assess stress distribution and deformation under load in scoliotic spines. In brace treatment, FE analysis can evaluate how different force configurations and magnitudes affect scoliosis correction and brace performance, leading to optimized designs that make braces lighter and more effective. Chou et al. [13] found that rotating the thoracic pad by 20 degrees dorsally in a Boston brace provides optimal corrective effects; Ali et al. [14] demonstrated that using a soft brace instead of a traditional rigid brace allows for spinal mobility while correcting, helping to reduce common side effects of rigid braces, such as muscle atrophy and skin issues. FE analysis can assess the therapeutic outcomes and potential complications of surgical treatment. For instance, Somtua et al. [15] studied the stress on pedicle screws and strain on vertebrae in patients with different Cobb angles undergoing surgical correction, finding that, when the Cobb angle exceeds 40 degrees, the vertebral strain may increase the risk of fractures. Currently, however, there is limited FE analysis research focused on the biomechanical effects on adjacent segments before and after surgery for AIS.

Previous FE analysis studies have typically used computed tomography (CT) or X-ray imaging to reconstruct the spinal morphology. CT-based models can accurately reconstruct the three-dimensional (3D) spine but have limitations due to high radiation exposure and the inability to reconstruct soft tissues like intervertebral disks. X-ray imaging, conversely, involves lower radiation exposure and allows for 3D reconstruction through parameterization. However, parameterized models often struggle to replicate the vertebral deformities seen in severe scoliosis cases.

To investigate the biomechanical effects in adjacent joints before and after surgical treatment in AIS patients, and given that current FE models based on X-ray images cannot accurately simulate vertebral deformities in severe scoliosis, this study aims to develop a patient-specific parameterized FE model for AIS patients using X-ray imaging, incorporate coronal plane vertebral deformation to simulate pre- and postoperative biomechanics, and analyze the ROM and stress distribution changes in adjacent segments post-operatively.

## 2. Materials and Methods

### 2.1. Study Subject

This study recruited a patient with Lenke type 5 AIS and a Cobb angle of 52 degrees, who was eligible for posterior spinal instrumentation surgery. The study was approved by the Institutional Review Board of National Yang-Ming University (#YM111055F), and the participant provided written informed consent before enrollment. Based on the patient’s biplanar X-ray images, a case-specific, parameterized FE model was constructed to simulate the changes in ROM and stress distribution pre- and post-surgery.

### 2.2. Parameterized Finite Element Model

The parameterized FE model was based on a lumbar spine model that has been validated and used in previous studies [16,17,18,19] and incorporated coronal plane vertebral deformation. The parameterization method followed the model developed by Chou et al. [13] and was implemented using ANSYS 14.5 with the parametric design language (APDL) (ANSYS, Inc., Houston, TX, USA). The model consists of the spinal section and implanted instrumentation. The spinal section includes 18 vertebrae from L5 to C7, 17 intervertebral disks, and associated ligaments. Each vertebra comprises cortical bone, cancellous bone, and posterior elements. The intervertebral disks include the nucleus pulposus, annulus fibrosus, disk fibers, and endplates. At the same time, the ligaments consist of anterior and posterior longitudinal ligaments, the interspinous ligament, the supraspinous ligament, the ligamentum flavum, the intertransverse ligament, and the facet joint capsules. Vertebral bodies were modeled using Solid45 elements, the nucleus pulposus with Fluid80 elements, and the annulus fibrosus and endplates with Solid45 elements, while the Link10 elements represented disk fibers and ligaments. The spinal model comprises 17,439 elements and 17,043 nodes. The material properties of the scoliotic FE model were listed in Table 1. The lumbar spine model has been validated in previous studies [17,20], showing stiffer behavior during flexion compared with in vitro cadaveric testing, with ROM values being 4° lower than those described in Rohlmann’s in vitro study. Additionally, the model exhibited greater stiffness in extension and rotation, although the differences remained within 2°. The discrepancies between in vitro testing and FE simulations were within one standard deviation.

**Figure 1:**
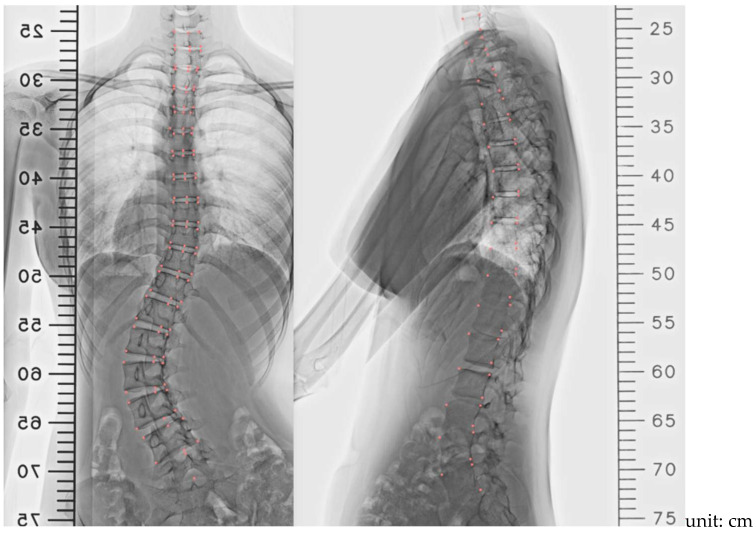
X-ray image parameters were obtained with a 3D Slicer in PA view and lateral view. Note: Pink markers indicate sampling points.

**Figure 2:**
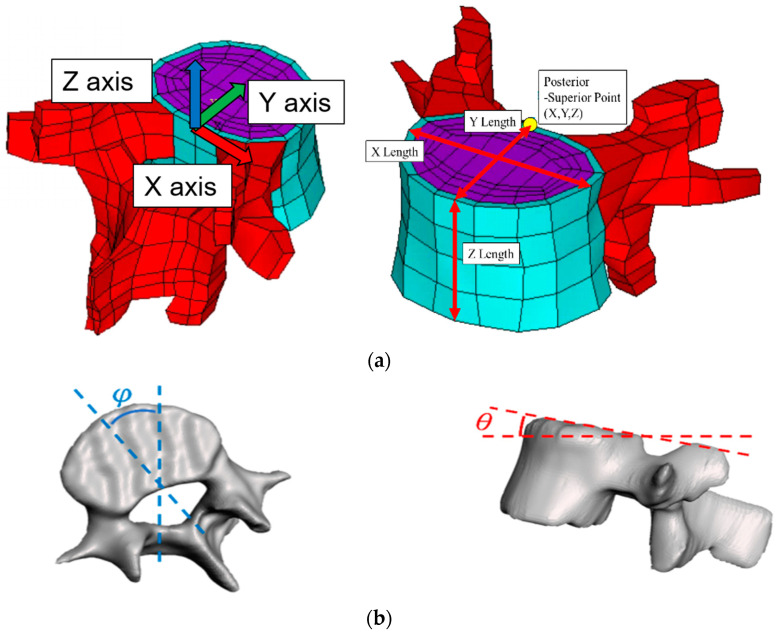

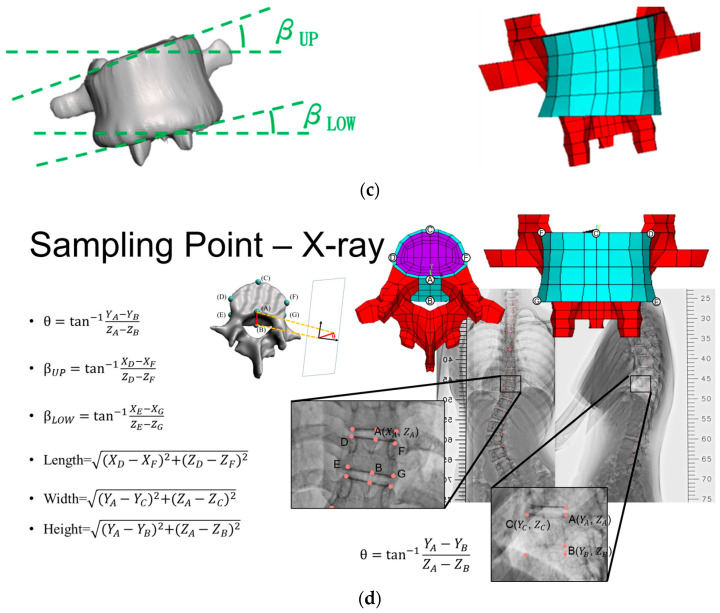
Adjustable spine parameters: (**a**) Coordinates of the superior posterior point and the vertebral body lengths along the three axes. (**b**) Sagittal tilt angle of the vertebral body and transverse tilt angle of the vertebral body. (**c**) Coronal tilt angles of superior and inferior endplates. (**d**) Calculation of angle and length from nodal locations. Note: 1. Sampling points included A, B, C, D, E, F, and G; 2. X, Y, Z indicate the coordinates.

The parameterized FE model of the spine was constructed using biplanar X-ray images, including posterior–anterior (PA) and lateral views, obtained with 3D Slicer version 4.10.2 [21]. The entire spine was reconstructed using a lumbar vertebral body L5. The relative location and angle of the whole spine were determined from two X-ray images. Sampling coordinates X0, Y0, and Z0 were extracted from the images (Figure 1) and transformed into FE model coordinates X, Y, and Z using Euler angle rotations in the Y-X-Z order (Equation (1)). The vertebral rotation was defined using the Nash–Moe method and judged by the location of the pedicle [22]. Adjustable parameters in the FE model include the following:

1. Coordinates of the superior posterior points of each vertebral body and vertebral body length along the three axes (Figure 2a);
2. Sagittal (θ) and transverse (φ) tilt angles of each vertebral body (Figure 2b);
3. Coronal tilt angles (β) of the superior and inferior endplates and their FE model (Figure 2c).

**Figure 3:**
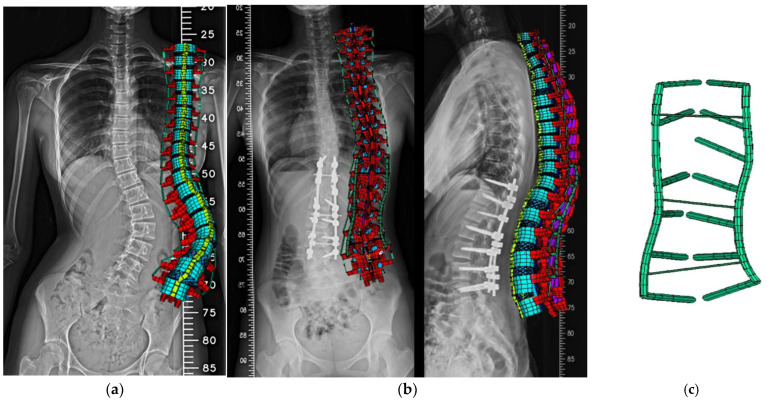
Posterior views of the case-specific FE model: (**a**) pre-op, (**b**) post-op, (**c**) instrumentation.

The user could input the above parameters to create the FE model of the entire spine automatically. The pre- (Figure 3a) and postoperative (Figure 3b) FE models, along with the instrumentation model (Figure 3c), were then established. Instrumentation includes pedicle screws and connecting rods. In the post-operative model, pedicle screws were placed bilaterally from T10 to T11 and L1 to L4, and unilaterally at T12. Two rods were used to secure screws on each side, with transverse connectors linking rods at T11, L2, and L3. The lumbar screws were 6.6 mm in diameter, the thoracic screws and rods were 5.5 mm, and the transverse connectors were 2 mm. Pedicle screws and rods were modeled using Beam188 elements, with 99 elements and 98 nodes. All materials were assumed to be linear and isotropic, with parameters based on previous studies [20,23].

(1) $$\left\{\begin{array}{c} \left[\begin{array}{c} X_{1}\\ Y_{1}\\ Z_{1}\\ 1 \end{array}\right]=\left[\begin{array}{cccc} \cos\beta & 0 & \sin\beta & 0\\ 0 & 1 & 0 & 0\\ -\sin\beta & 0 & \cos\beta & 0\\ 0 & 0 & 0 & 1 \end{array}\right]\left[\begin{array}{c} X_{0}\\ Y_{0}\\ Z_{0}\\ 1 \end{array}\right]\\ \left[\begin{array}{c} X_{2}\\ Y_{2}\\ Z_{2}\\ 1 \end{array}\right]=\left[\begin{array}{cccc} 1 & 0 & 0 & 0\\ 0 & \cos\theta & -\sin\theta & 0\\ 0 & \sin\theta & \cos\theta & 0\\ 0 & 0 & 0 & 1 \end{array}\right]\left[\begin{array}{c} X_{1}\\ Y_{1}\\ Z_{1}\\ 1 \end{array}\right]\\ \left[\begin{array}{c} X\\ Y\\ Z\\ 1 \end{array}\right]=\left[\begin{array}{cccc} \cos\varphi & -\sin\varphi & 0 & 0\\ \sin\varphi & \cos\varphi & 0 & 0\\ 0 & 0 & 1 & 0\\ 0 & 0 & 0 & 1 \end{array}\right]\left[\begin{array}{c} X_{2}\\ Y_{2}\\ Z_{2}\\ 1 \end{array}\right] \end{array}\right\}$$

Note:

1. X0, Y0, and Z0: original nodal coordinate in the vertebral body L5
2. X1, Y1, and Z1: new nodal coordinate in a new vertebral body after rotating an angle of β (frontal plane)
3. X2, Y2, and Z2: new nodal coordinate in a new vertebral body after rotating an angle of θ (sagittal plane)
4. X, Y, and Z: new nodal coordinate in a new vertebral body after rotating an angle of φ (transverse plane)
5. A total of 782 nodes were in the vertebral body L5

### 2.3. Model Validation

**Figure 4:**
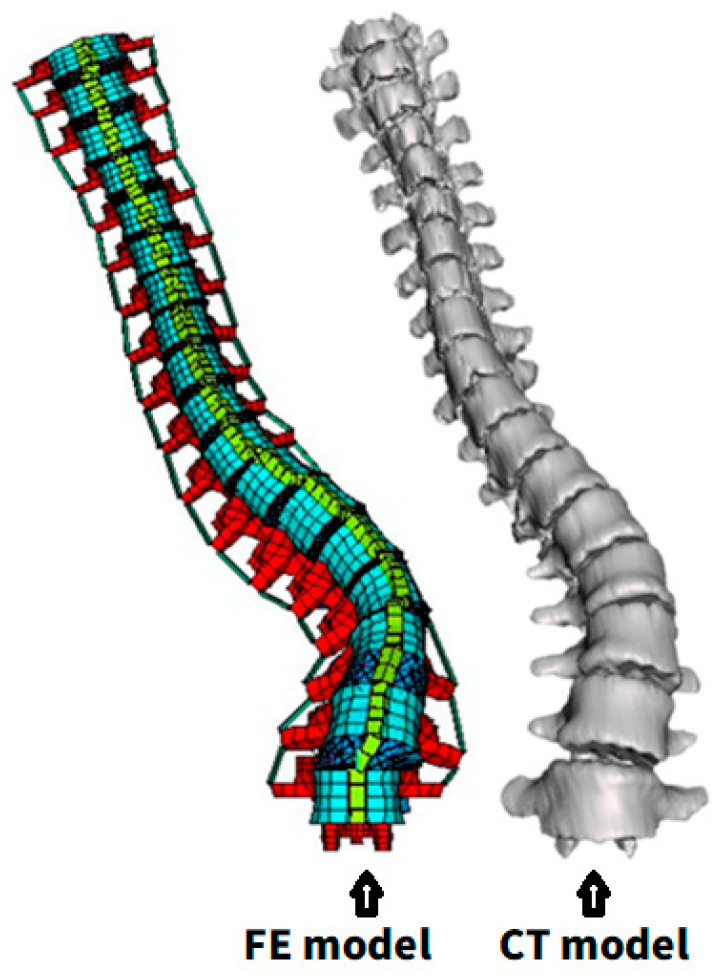
Comparison between FE- and CT-based reconstructed 3D model.

To ensure the geometric accuracy of the model, the FE model constructed from X-ray images was compared with the patient’s CT images. CT imaging provides detailed 3D spinal data, allowing for model accuracy assessment. Using a software 3D Slicer 4.10.2, a 3D reconstruction of the CT images was performed, and the geometries were compared with the X-ray-derived model (Figure 4). The spatial coordinate error of each vertebra was calculated and presented as the mean absolute error (MAE) (Equation (2)).

**Figure 5:**
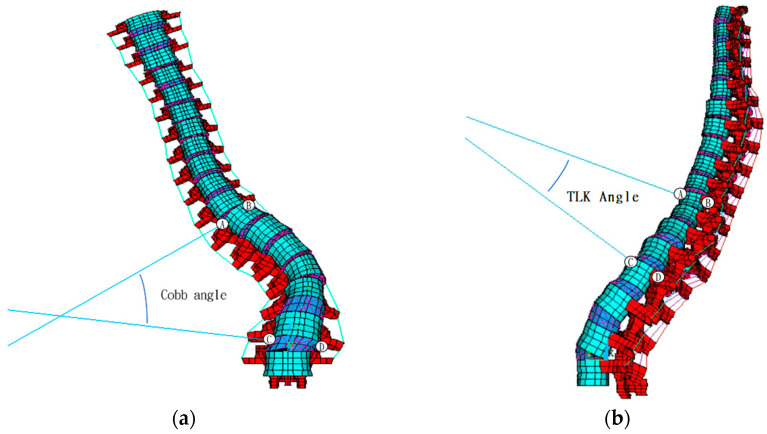
Calculation of FE model’s (**a**) Cobb angle and (**b**) TLK angle. **Note:** A, B, C and D indicate the sampling point to measure the Cobb’s and TLK angles.

To verify the model’s angular accuracy, the Cobb (Figure 5a) and thoracolumbar kyphosis (TLK) angles (Figure 5b), measured from the FE model, were compared with values obtained from imaging.

(2) $$\mathrm{MAE}=\sum_{i=1}^{18}\left|{FE\;model}_{i}-{CT}_{i}\right|$$

Note:

1. i = 1~18 indicates each vertebral body from L5 to C7.
2. MAE indicates the mean absolute error between the FE model and the CT images.

### 2.4. Boundary and Loading Conditions

The inferior end of the L5 segment was fixed to simulate the patient’s actual movements, representing lower body support. A flexion moment was applied to the C7 segment to replicate the loading conditions during forward bending. A flexion moment of 10 Nm was used in the preoperative spine model [17]. Incremental force was applied to the C7 vertebra for the postoperative model to control the overall spinal angle, ensuring that it matched the preoperative model during flexion [24]. FE analysis was performed on the pre- and postoperative models with the same flexion moment. Analysis parameters included the overall stress distribution, range of motion (ROM), annulus fibrosus stress, and endplate stress changes before and after surgery.

## 3. Results

### 3.1. Model Validation

**Figure 6:**
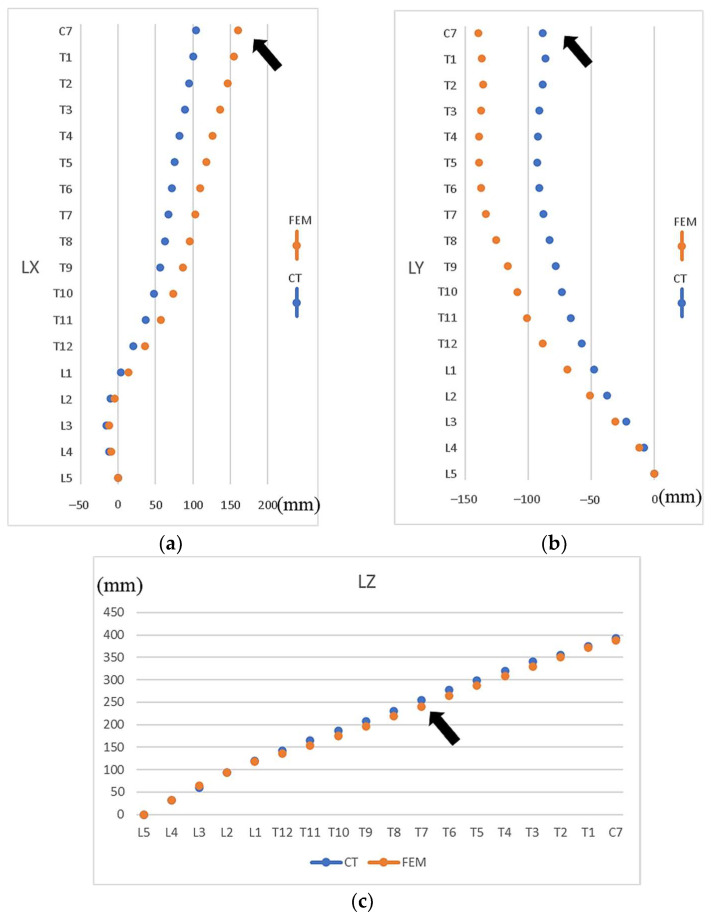
Differences in vertebral locations from the bottom vertebral body L5 to upper vertebral body C7 between FE model and CT images. (**a**) X-axis coordinates (ML direction), (**b**) Y-axis coordinates (AP direction), (**c**) Z-axis coordinates (vertical). Note: Arrows indicate the vertebrae with the most significant discrepancies. LX, LY, and LZ represent the X-, Y-, and Z-coordinates of the vertebral bodies.

The preoperative parameterized FE model closely resembled the CT images in overall shape. The MAE of the X-coordinate of vertebral bodies (median lateral direction) was 28.8 mm, with the most significant discrepancy being found at C7 (Figure 6a); for the Y-coordinate (anterior–posterior direction), the MAE was 33.7 mm, with the most significant error occurring at C7 (Figure 6b); for the Z-coordinate (vertical direction), the MAE was 7.4 mm, with the most considerable discrepancy being observed at T7 (Figure 6c).

The Cobb angle measured by the FE model was 45.5° preoperatively and 13.9° postoperatively, while X-ray measurements yielded 52° and 11°, respectively, indicating a discrepancy of less than 6.5° between the two methods.

The TLK angle measured using the FE model was 20.2° preoperatively and 10.0° postoperatively, while X-ray measurements showed 15.3° and 4.6°, respectively, leading to a discrepancy of less than 5.4° between the two methods.

### 3.2. Biomechanical Analysis

#### 3.2.1. Segmental ROM

During forward bending, the ROM of the surgically treated segments (T10-L4) post-surgery decreased to 21.1% of the preoperative ROM (Table 2). The ROM of the cranial adjacent segment (T9-T10) increased by approximately 1.35 times compared with pre-surgery, while the caudal adjacent segment (L4-L5) decreased to 39.2% of the preoperative ROM. The ROM of other cranial segments (C7-T10) increased to about 1.7 times the preoperative value.

#### 3.2.2. Maximum Stress in Endplate and Annulus Fibrosus

**Figure 7:**
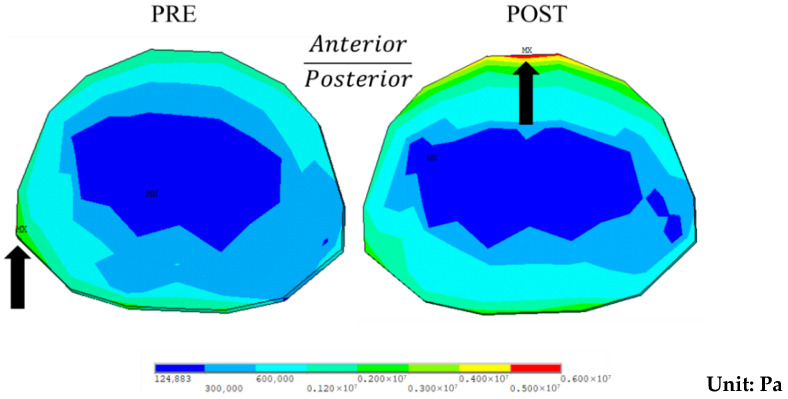
Stress distribution at the superior endplate of T10 during flexion preoperatively (PRE) and postoperatively (POST). Note: Black arrows indicate areas of high stress.

For the endplate, the maximum stress at the superior endplate of T10 increased from 3310 kPa preoperatively to 5770 kPa postoperatively during flexion. The stress concentration shifted from the concave side preoperatively to the ventral side postoperatively (Figure 7). For the inferior endplate of L4, the maximum stress decreased from 6430 kPa preoperatively to 1710 kPa postoperatively, about 26.6% of the preoperative level, with stress being concentrated on the dorsal side in both cases.

For the annulus fibrosus, the maximum stress in the cranial adjacent segment (T9-T10) increased from 634 kPa preoperatively to 1460 kPa postoperatively, approximately 2.3 times the preoperative level, with the stress concentrating on the ventral side. In the caudal adjacent segment (L4-L5), the maximum stress decreased from 1670 kPa preoperatively to 416 kPa postoperatively, about 24.9% of the preoperative level, with the stress being concentrated on the dorsal side.

#### 3.2.3. Overall Stress Distribution

**Figure 8:**
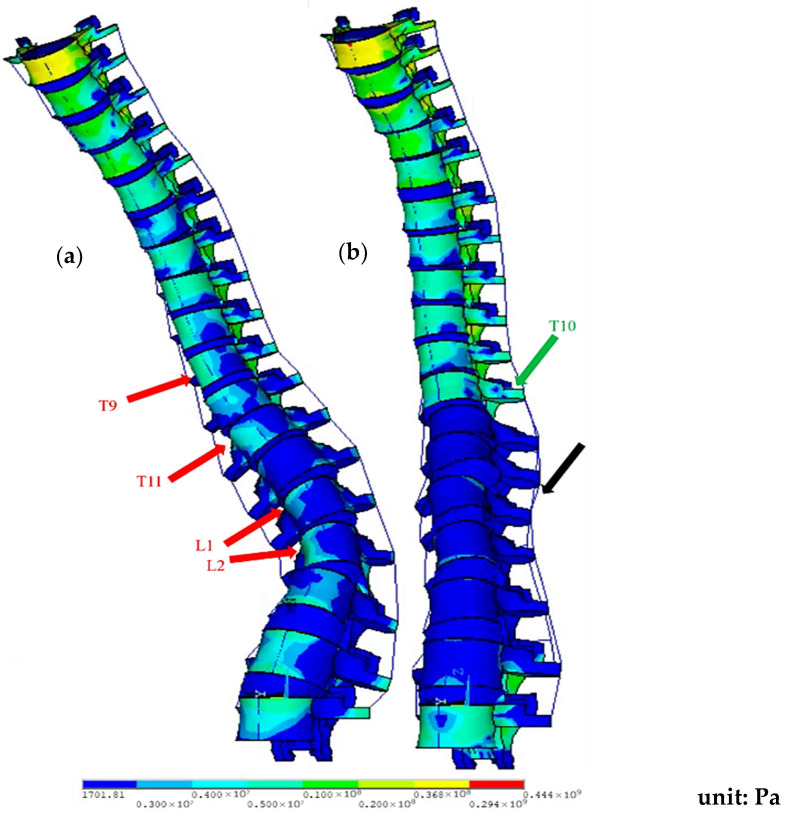
Stress distribution of the spine model during flexion: (**a**) pre-op; (**b**) post-op (fixed at T10-L4). Note: Red arrows show the pre-op stress concentration on the concave side of the curve; black arrows indicate reduced stress in the surgical segments; and green arrows indicate higher stress at T10.

The movement and stress distribution in the complete spinal model during flexion are illustrated in Figure 8. In the preoperative model, stress is concentrated on the concave side of the scoliotic curve, as indicated by the red arrows. In the post-operative model, most surgically treated segments (T10-L4) exhibit significantly reduced stress, except T10, which experiences higher stress levels.

## 4. Discussion

### 4.1. Model Reconstruction

In previous studies, FE models have been constructed based on CT and X-ray images. Using CT images to build models presents several difficulties, such as radiation exposure and accurately modeling the intervertebral disk. For example, Wei et al. [24] used reverse engineering software to construct the disk, and Somtua et al. [16] utilized CAD software like SolidWorks to build the disk. In contrast, models constructed from X-ray images struggle to capture vertebral shapes accurately. For instance, Ali et al. [15] used X-ray images and beam elements to build an FE model, representing each vertebral body with a single beam element, which cannot depict the deformations of the vertebral body that are often seen in severe scoliosis cases.

This study developed a parameterized FE model based on X-ray images to accurately represent vertebral body deformation, thus avoiding the high radiation dose associated with CT imaging while overcoming the limitations of X-ray in capturing vertebral deformities. This model benefits patients with AIS requiring surgical intervention and those with milder scoliosis (with Cobb angles below 40 degrees), who may only need bracing or physical therapy. Since CT scans are not routine for these patients, constructing high-precision models from CT images is challenging. Using low-radiation X-ray images, the parameterized model can reconstruct an FE model that captures vertebral body deformation, supporting biomechanical analysis of scoliosis and the design of corrective devices such as the Boston brace.

### 4.2. Model Validation

The Cobb angle calculated by the FE model and the X-ray measurement differed by 6.5 degrees preoperative and 2.9 degrees postoperative. A study [25] found that the Cobb angle measured by different observers may differ by 2.8 to 10 degrees within a 95% confidence interval. Therefore, the error in this study is acceptable. As for the reason why the Cobb angle measured by X-ray pre-operative is larger than the result calculated by the FE model, but the result measured by X-ray postoperative is smaller, there may be two reasons: the projection of the 3D angle and the vector skew. When the angle in 3D space is projected onto the 2D plane, its size may change, and it may become larger or smaller depending on the projection angle; while when using the vector angle to calculate the angle in space, if the vector is not coplanar but skewed, it may be affected by the angle between different planes. Therefore, the vector angle is affected not only by the coronal plane angle but also by the transverse plane angle, resulting in an angle larger than expected.

The TLK angle measured from the FE model also deviated by only 5.4 degrees from the X-ray measurements. Biomechanically, FE analysis revealed that post-operative flexion increased the ROM and maximum stress in the cranial adjacent segments, while the ROM and stress in the caudal adjacent segments decreased.

In terms of vertebral location, the MAE values of 28 mm (ML direction) and 34 mm (AP direction) were slightly higher, despite the subject having a spine length of 390 mm (C7-L5). Two reasons were attributed to these errors. First, the discrepancies between the FE model and CT images are primarily due to differences in the subject’s posture during the imaging process. The FE model parameters were derived from standing, weight-bearing X-ray images, whereas the CT images were taken with the subject in a supine position [26]. Second, the error may be due to the measurement of the vertebral body L5. All vertebral bodies were generated based on the vertebral body L5. We found that inaccurately digitizing the location of the L5 vertebral body in X-ray images would result in more errors in the FE scoliotic model. Therefore, the user should carefully digitize the location of the vertebral body L5 in X-ray images.

Additionally, the errors in the Cobb and TLK angle calculations from the model compared with X-ray measurements arose mainly from the 3D-to-2D projection process, where the skew in vectors may also introduce measurement errors. Previous studies have reported discrepancies between Cobb angle measurements from 3D and 2D images [27,28], with several studies also finding that inter-observer variation in Cobb angle measurements can differ by 2.8 to 10 degrees within a 95% confidence interval [29], making the errors in this study acceptable.

Although there are some errors in the FE scoliotic model, an experienced technician could create a scoliotic FE model within 3 h using X-ray images, and the FE model could be rapidly constructed for clinical application.

### 4.3. Biomechanical Analysis

This study found that the ROM of cranial unfused segments increased to approximately 1.7 times the preoperative level for the segmental ROM. In contrast, the ROM of caudal unfused segments decreased to around 39.2% of the preoperative level. This study found that the mobility of the unfused cranial segment accounted for 52.2% of the total mobility preoperatively and 87.7% postoperatively, an increase of 35.5%. Ensberg et al.’s study [27] found that the mobility of the unfused cranial segment accounted for 38.5% of the total mobility preoperatively and 69.0% postoperatively, an increase of 30.5%. It is evident that in both studies, the unfused cranial segment compensated for most of the reduced ROM in the fused segment.

Regarding endplate stress, the maximum stress of caudal adjacent segments decreased post-surgery. In contrast, cranial adjacent segments experienced an increase in maximum stress, with a more balanced stress distribution. This outcome may be related to the increased ROM in the cranial segments and the decreased ROM in the caudal segments after surgery; furthermore, Akazawa et al. [5,6,7] suggest that Modic changes observed in patients with AIS may result from the concentration of stress on the concave side of the scoliotic curve. Therefore, the even stress distribution post-surgery might help reduce the likelihood of Modic changes.

For annulus fibrosus stress, the maximum stress of cranial adjacent segments increased post-operatively, while that in caudal adjacent segments decreased. This finding is consistent with the study by Nohara et al. [28], which, after a ten-year follow-up, found that AIS patients who had been treated with corrective surgery had significantly lower rates of disk degeneration at the L3-4 and L4-5 segments than untreated patients, with reductions of 35.1% and 33.4%, respectively. This may suggest that surgical treatment for AIS reduces the maximum stress on the intervertebral disks in caudal adjacent segments, lowering the likelihood of disk degeneration.

### 4.4. Study Limitations and Assumptions

This study has several limitations: (1) the quality of the X-ray images may affect the accuracy of the FE model; (2) the shape of the FE model is based on scaling and deforming adult L5 vertebrae in various directions, which may not accurately reflect all anatomical features of the human spine, such as the facet joint orientation; (3) since contact element was not assigned to the facet joints, only flexion movements—which impact the facet joints minimally—were analyzed, excluding extension, lateral bending, and axial rotation; (4) this study only measured the ROM in the sagittal plane, without accounting for the coupling effect in scoliosis; (5) material properties of the vertebral body and disc in the FE model were assumed to be linear and isotropic, which the isotropy of the vertebral body and disc would result in a stiffer behavior of the lumbar spine than anisotropy of the vertebral body and disc [13,20]; the more realistic material properties should be added to the FE model in future studies [30,31]; (6) the study was based on data from a single Lenke type 5 AIS case, limiting the generalizability to other AIS types or broader patient populations; (7) Since the study aimed to generate an FE model using APDL codes rapidly, the number of nodes in the vertebral body L5 was fixed. As a result, the mesh convergence was not conducted in the study because it would alter the nodal numbers of the original vertebral body. Therefore, the coarse mesh might lower the accuracy of the FE calculation.

## 5. Conclusions

This study successfully used biplanar X-ray images to obtain vertebral coordinates, size, and tilt angles to construct a patient-specific parameterized FE model. The model also incorporated coronal plane vertebral deformation to represent severe scoliosis accurately. The differences in Cobb and TLK angles between the model and X-ray measurements were within 6.5 and 5.4 degrees, respectively. Using this model, flexion—a common spinal movement—was simulated, and the ROM, endplate stress, and disk stress in adjacent segments were analyzed. The findings suggest that surgical treatment for patients with Lenke type 5 AIS may reduce the ROM of caudal adjacent segments to about 40% of the preoperative level, with maximum endplate stress decreasing to approximately 27% and maximum disk stress to about 25%.

## Figures and Tables

**Table 1:** Material properties of the scoliotic FE model [17].

Material	Young’s Modulus (MPa)	Poisson’s Ratio	Area (mm^2^)
**Bone**			
Cortical	12,000	0.3	-
Cancellous	100	0.2	-
Posterior element	3500	0.25	-
**Disc**			
Nucleus pulposus	1	0.499	-
Ground Substance	4.2	0.46	-
Annulus Fibers	175	0.4	
Endplate	24	0.4	-
**Ligament**			
ALL	7.8		24
PLL	10		14.4
TL	10		3.6
LF	15		40
ISL	10		26
SSL	8		23
CL	7.5		30

ALL, anterior longitudinal ligament; CL, capsular ligament; ISL, interspinous ligament; LF, ligamentum flavum; PLL, posterior longitudinal ligament; SSL, supraspinous ligament; TL, transverse ligament.

**Table 2:** Comparison of ROM and the maximum von Mise stress in adjacent segments.

	**Pre-Op**	**Post-Op**
ROM (°)		
C7-T10	21.52	36.31
T9-T10	1.71	2.31
T10-L4	14.53	3.06
L4-L5	5.21	2.04
Total	41.26	41.41
	**Max Endplate Stress (MPa)**	
T10	3.31	5.77
L4	6.43	1.71
	**Max Annulus Fibrosus Stress (MPa)**	
T9-T10	0.63	1.46
L4-L5	1.67	0.42

Note: The surgical segments are from T10 to L4; the adjacent segments are T9-T10 and L4-L5.

## Data Availability

The original contributions presented in the study are included in the article, further inquiries can be directed to the corresponding author.
